# To Contributors

**Published:** 1886-08

**Authors:** 


### TO CONTRIBUTORS.

We are again obliged to ask for consideration at the hands of contributors. So much of excellent matter has been offered that it is impossible to print it all without delay. Each month all that the number will contain is printed, but still many instructive articles are necessarily held over. Sometimes the best of contributions are withheld a month, because other articles on the same subject are in type. We can assure our correspondents that their favors are valued very highly, and it is a matter of pride with us that, so great a favorite is the journal, at all times we have an abundance of good material.

